# Estimating past hepatitis C infection risk from reported risk factor histories: implications for imputing age of infection and modeling fibrosis progression

**DOI:** 10.1186/1471-2334-7-145

**Published:** 2007-12-10

**Authors:** Peter Bacchetti, Phyllis C Tien, Eric C Seaberg, Thomas R O'Brien, Michael H Augenbraun, Alex H Kral, Michael P Busch, Brian R Edlin

**Affiliations:** 1 Box 0560, Department of Epidemiology and Biostatistics, University of California, San Francisco, CA 94143, USA; 2 Department of Medicine, University of California, San Francisco and Infectious Disease Section, 111W, Veterans Affairs Medical Center, San Francisco, CA 94121, USA; 3 Department of Epidemiology, Johns Hopkins University, Bloomberg School of Public Health, Baltimore, MD, USA; 4 Division of Cancer Epidemiology and Genetics, National Cancer Institute, Bethesda, MD, USA; 5 SUNY Downstate Medical Center, Division of Infectious Diseases, 450 Clarkson Avenue, Box 56, Brooklyn, NY 11203, USA; 6 RTI International, San Francisco Regional Office; and Department of Family and Community Medicine, University of California, San Francisco, CA 94143, USA; 7 Blood Systems Research Institute, San Francisco and the Department of Laboratory Medicine, University of California, San Francisco, CA 94143, USA; 8 Center for the Study of Hepatitis C, Weill Medical College of Cornell University, 411 East 69th St., Room KB-319, New York, NY 10021, USA; 9 Department of Family and Community Medicine, University of California, San Francisco, CA 94143, USA

## Abstract

**Background:**

Chronic hepatitis C virus infection is prevalent and often causes hepatic fibrosis, which can progress to cirrhosis and cause liver cancer or liver failure. Study of fibrosis progression often relies on imputing the time of infection, often as the reported age of first injection drug use. We sought to examine the accuracy of such imputation and implications for modeling factors that influence progression rates.

**Methods:**

We analyzed cross-sectional data on hepatitis C antibody status and reported risk factor histories from two large studies, the Women's Interagency HIV Study and the Urban Health Study, using modern survival analysis methods for current status data to model past infection risk year by year. We compared fitted distributions of past infection risk to reported age of first injection drug use.

**Results:**

Although injection drug use appeared to be a very strong risk factor, models for both studies showed that many subjects had considerable probability of having been infected substantially before or after their reported age of first injection drug use. Persons reporting younger age of first injection drug use were more likely to have been infected after, and persons reporting older age of first injection drug use were more likely to have been infected before.

**Conclusion:**

In cross-sectional studies of fibrosis progression where date of HCV infection is estimated from risk factor histories, modern methods such as multiple imputation should be used to account for the substantial uncertainty about when infection occurred. The models presented here can provide the inputs needed by such methods. Using reported age of first injection drug use as the time of infection in studies of fibrosis progression is likely to produce a spuriously strong association of younger age of infection with slower rate of progression.

## Background

Chronic hepatitis C virus (HCV) infection is prevalent both in the United States, with perhaps 3 million persons infected [1], and worldwide [2]. It often causes progressive hepatic fibrosis. This can eventually become cirrhosis, causing hepatocellular carcinoma, liver failure, a need for liver transplant, or death. Fibrosis measured by liver biopsy is believed to provide the most accurate measurement of the extent of liver damage [3]. Considerable effort has been devoted to estimating risk factors for infection [4-13], as well as rates of progression through fibrosis stages to cirrhosis [14-21]. Studies of HCV progression often utilize liver biopsies from persons who may have been originally infected many years or decades earlier. The exact time of infection is often unknown, so the subjects' self-reported risk factor histories are used to impute a presumed time of infection. A common practice is to assume that infection occurred at the time of first reported injection drug use (IDU) [14,19,22]. Studies of infection risk factors support this practice to a limited extent by showing that IDU is a strong risk factor [4,5,7,8,12]. Here, we assess in more detail how accurate such imputation is likely to be. We utilize cross-sectional HCV antibody status data and self-reported risk-factor histories, similar to the situation in progression studies where time of infection must be imputed. In contrast to the usual statistical modeling in risk factor studies, which utilizes logistic regression with potential risk factors modeled as fixed covariates, we use survival analysis methods with time-varying covariates. This is possible even though every observation is either left-censored (infection occurred at some unknown time in the past) or right censored (infection has not yet occurred). An advantage of this approach is that it permits reconstruction of past risk year by year, which facilitates assessment of possible biases or inaccuracies in the usual imputation strategy, along with the implications for modeling of factors that influence fibrosis progression.

## Methods

### Study population

We analyzed data from two large studies that performed HCV antibody (anti-HCV) testing and collected subject reports concerning history of IDU. The Women's Interagency HIV Study (WIHS), as previously described [23,24], includes women with or at risk for HIV infection. This study performed anti-HCV testing of initial participants, mainly in 1994–1995, and additional participants who were recruited in 2001–2002. The results used here were obtained using a second generation HCV enzyme immunoassay for most subjects, with a few of the newer recruits having data from a third generation test. The Urban Health Study (UHS) was composed of street-recruited injection drug users in the San Francisco Bay Area and has also been described previously [25-27]. Participants included here were recruited from 1987 to 2002, and second generation anti-HCV testing was performed for specimens from 1987 and from 1998–2002, as well as the initial specimens regardless of year for all subjects who reported IDU duration of < 10 years. For our analyses of both studies, we included only the first anti-HCV result from each participant and did not include any subsequent longitudinal follow up. This was to reduce cohort participation bias [12,28,29], and to approximate the common situation in cross-sectional studies of patients presenting with chronic HCV infection with no prior HCV test results. We also required that subjects have valid data on: sex; race; HIV antibody; ages of first and last IDU, if any; age and calendar year of HCV test; and for those with a history of IDU, whether they typically injected every day. For UHS, we assumed that subject's reported current injection frequency was typical of their entire IDU history because no other information was available about frequency of injection. WIHS subjects were asked to report a single typical frequency for all the time when they were injecting. Histories of needle sharing practices were not collected in either study. Because needle sharing behavior may have changed substantially over time due to the emergence of HIV and subsequent efforts to prevent its spread, current sharing would not be an adequate surrogate for past practices; we therefore did not assess this risk factor. Data on blood transfusion history was not required for inclusion in our analyses because it was only partially ascertained in WIHS (transfusions in the years 1975–1985, only among those recruited in 1993–94) and was not collected in UHS.

### Statistical methods

The available data indicate only whether or not each subject was infected with HCV at some point in the past, a form of information known in the statistical literature as current status data [30-33]. We used maximum full likelihood to fit parameters of discrete-time survival models, using year of age as the time scale. Beginning with age 1 (where risk was very small in all models), the log-odds of HCV infection given no previous infection was modeled as a linear function of covariates that pertained to that year, including age, calendar year, and whether the subject reported using injection drugs during that year. The fitted log-odds were then converted to hazards and combined across years to obtain a predicted probability of being HCV seropositive at the age tested. The NLMIXED procedure in the SAS statistical package (version 9.1, SAS Institute, Cary, NC) was used to obtain the parameter estimates that maximized the resulting likelihood, along with their confidence intervals (CI) and p-values. This approach is similar to Cox's discrete-time partial likelihood model [34], except that the role of time (in this case age) is modeled instead of being conditioned out in a partial likelihood. It can also be considered a form of pooled logistic regression [35] with a parsimonious model for the effect of time instead of an unstructured model. Estimated effects of covariates are presented as odds ratios (OR's). We provide in Additional File 1 the SAS NLMIXED code for fitting one of the models presented below.

Because HCV risk associated with IDU may vary depending on duration of IDU [7,12,36], we defined separate time-varying indicator variables for the reported first year of use, the second or third year, and the fourth or greater year. In defining the hazard at each age, we assumed that IDU began in the middle of the reported age of first use; instead assuming that IDU began at the beginning produced qualitatively similar results, but may be a less accurate assumption. We also examined further breakdown of duration into fourth to tenth year versus 11^th ^or greater year. For the time-varying numeric covariates age and calendar year, we examined linear models, quadratic models, and more flexible models based on parametric cubic splines defined by the ns() function in S-Plus (Insightful Corporation, Seattle, WA), choosing a model by likelihood ratio testing of nested alternatives. We examined plausible interactions one at a time by adding the product of the two predictors to the model with main effects only. To limit the complexity of interactions with reported IDU, we assumed common interactions for the reported first, second-third, and fourth or greater years of IDU.

We used the fitted multivariate models to calculate estimated distributions of age at HCV infection given each subject's age at first positive HCV antibody test and reported risk factor profile. For each subject and each possible age of infection, this produced a probability that infection occurred at that age. To compare these to the usual imputation of age at HCV infection as the reported age of first IDU, we calculated the mean age at infection from the estimated probabilities and calculated the usual imputation's bias as the age of first IDU minus this mean. We also calculated the probability that infection occurred at the reported age of first IDU or the next year, considering this to be the probability that the usual imputation would be reasonably accurate.

## Results

### Descriptive summaries

The characteristics of 2248 WIHS participants and 4623 UHS participants analyzed in this study are summarized in Table 1. Both studies contain sufficient numbers of HCV seropositive and seronegative subjects to permit analysis of risk factors, but there are substantial differences between the studies due to different target populations and inclusion criteria. Although WIHS enrolled 3766 women, IDU history was only assessed in enough detail for the present study 8 years after initial enrollment. At that time, 2318 women provided information on ages of IDU, if any, and 70 of these (3.0%) were excluded for lack of valid data on other key variables. Of 4734 potential UHS participants, 111 (2.3%) were excluded due to lack of valid data on key variables.

**Table 1:** Characteristics of WIHS and UHS subjects analyzed for this study.

	N in category (% of total)	
	
Characteristic	WIHS (N = 2248)	UHS (N = 4623)
HCV seronegative	1666 (74.1%)	576 (12.5%)
HCV seropositive	582 (25.9%)	4047 (87.5%)
		
Year of HCV test		
1987–1992	0 (0.0%)	572 (12.4%)
1993	29 (1.3%)	48 (1.0%)
1994	844 (37.5%)	47 (1.0%)
1995	306 (13.6%)	57 (1.2%)
1996	2 (0.1%)	62 (1.3%)
1997	4 (0.2%)	37 (0.8%)
1998	76 (3.4%)	862 (18.6%)
1999	15 (0.7%)	995 (21.5%)
2000	24 (1.1%)	904 (19.6%)
2001	635 (28.2%)	907 (19.6%)
2002	313 (13.9%)	132 (2.9%)
		
Age at HCV test		
< 20	58 (2.6%)	67 (1.4%)
20–29	593 (26.4%)	488 (10.6%)
30–39	1048 (46.6%)	1200 (26.0%)
40–49	486 (21.6%)	1925 (41.6%)
50–59	58 (2.6%)	810 (17.5%)
60+	5 (0.2%)	133 (2.9%)
		
Female	2248 (100%)	1409 (30.5%)
Male	0 (0%)	3214 (69.5%)
		
Race/Ethnicity		
Caucasian	275 (12.2%)	1684 (36.4%)
African American	1277 (56.8%)	2232 (48.3%)
Hispanic	615 (27.4%)	401 (8.7%)
Other	81 (3.6%)	306 (6.6%)
		
Area		
San Francisco	340 (15.1%)	4623 (100%)
Bronx	459 (20.4%)	0 (0%)
Brooklyn	429 (19.1%)	0 (0%)
Washington, DC	320 (14.2%)	0 (0%)
Los Angeles	397 (17.7%)	0 (0%)
Chicago	303 (13.5%)	0 (0%)
		
HIV-infected	1627 (72.4%)	787 (17.0%)
		
Ever IDU	522 (23.2%)	4623 (100%)
		
Age of First IDU		
Before 12	4 (0.8%)	123 (2.7%)
12–15	69 (13.2%)	972 (21.0%)
16–19	163 (31.2%)	1520 (32.9%)
20–29	227 (43.5%)	1416 (30.6%)
30–39	56 (10.7%)	464 (10.0%)
40+	3 (0.6%)	128 (2.8%)
		
Year of First IDU		
Before1960	2 (0.4%)	198 (4.3%)
1960–1969	78 (14.9%)	1174 (25.4%)
1970–1979	206 (39.5%)	1473 (31.9%)
1980–1989	173 (33.1%)	1015 (22.0%)
1990–1995	43 (8.2%)	459 (9.9%)
After1995	20 (3.8%)	304 (6.6%)
		
Daily IDU	341 (65.3%)	2992 (64.7%)

Table 2 shows detailed data on HCV prevalence by reported total duration of IDU in the two studies. This shows increasing prevalence of HCV seropositivity even at long durations, suggesting continuing risk among those who avoid infection early on. This also suggests that not everyone who is infected with HCV due to IDU is infected in the first year, as is commonly assumed.

**Table 2:** HCV Antibody status by duration of injection drug use (IDU) at time of testing.

	HCV seropositive/Total (%)	
	
Duration of IDU	WIHS	UHS
None	148/1726 (8.6%)	
1 year	5/9 (55.6%)	59/151 (39.1%)
2 years	5/8 (62.5%)	56/104 (53.8%)
3 years	6/11 (54.5%)	59/104 (56.7%)
4–5 years	13/20 (65.0%)	138/209 (66.0%)
6–7 years	15/21 (71.4%)	148/202 (73.3%)
8–10 years	33/43 (76.7%)	275/335 (82.1%)
11–15 years	84/102 (82.4%)	360/439 (82.0%)
16–20 years	91/109 (83.5%)	497/556 (89.4%)
21–30 years	167/184 (90.8%)	1345/1394 (96.5%)
> 30 years	15/15 (100%)	1110/1129 (98.3%)

### Models for HCV infection risk

**Figure 1:**
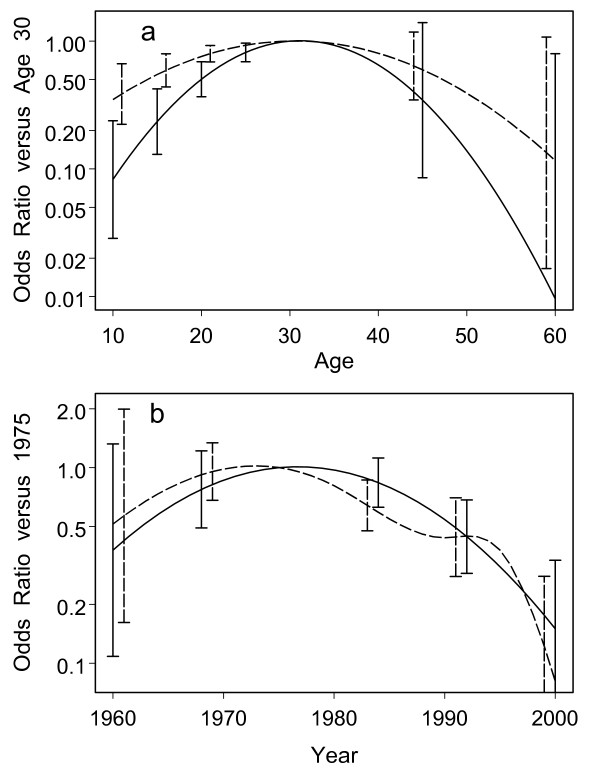
**Estimated effects of age and calendar year for the multivariate models of HCV infection risk**. WIHS: solid lines; UHS: dashed lines. Vertical bars are pointwise 95% confidence intervals. **a**) Estimated odds ratios for age. The reference age is 30, where the odds ratio is 1.0 by definition. **b**) Estimated odds ratios for calendar year. The reference year is 1975, where the odds ratio is 1.0 by definition.

Multivariate models of HCV risk for WIHS and UHS are shown in Table 3 and Figure 1. The estimated background risk for these models was 0.0051 (95% CI 0.0028 to 0.0091) for WIHS and 0.034 (95% CI 0.021 to 0.055) for UHS. This is the fitted probability of being infected with HCV over the course of a year at age 30 in 1975 for a previously HCV-uninfected, (reportedly) non-injecting, HIV-uninfected Caucasian female in the San Francisco area. Fitted probabilities for other situations and types of subjects are obtained by applying the OR's shown in Table 3 and Figure 1 to these background rates. Both cohorts produced some qualitatively similar results, including decreased risk at younger ages and more recent calendar years, as well as highest risk in the reported first year of IDU. Despite these similarities, there were too many quantitatively substantial differences to permit a simple model of both studies pooled. These include the background risk when not injecting, the shape of the drop in risk as reported duration of IDU increases, the role of daily IDU, the strength of the influence of age, and racial/ethnic associations. The model shown in Figure 1b for UHS is a parametric spline with knots at 1980, 1990, and 1995, because this provided an improved fit to the data compared to a quadratic model (p = 0.0028), even though its overall shape is roughly quadratic. The other curves in Figure 1 are all quadratic, because these improved substantially over linear models (WIHS p = 0.0003 for age and p = 0.0054 for calendar year; UHS p = 0.018 for age), but parametric splines with up to 4 parameters did not appear to substantially improve the fits further (all p > 0.22).

**Table 3:** Multivariate models of HCV infection risk.

	Women's Interagency HIV Study			San Francisco Urban Health Study		
		
Variable	Odds Ratio	95% CI	p	Odds Ratio	95% CI	p
Injection drug use (versus not injecting)						
1st year of use	310	163 to 588	< 0.0001	61	21 to 177	< 0.0001
2^nd ^& 3^rd ^year	163	65 to 404	< 0.0001	9.8	1.94 to 50	0.0058
4^th ^or later year	7.2	2.1 to 24.2	0.0015	5.2	3.2 to 8.4	< 0.0001
Daily IDU (versus less frequent IDU)	1.14	0.73 to 1.77	0.57	1.59	1.40 to 1.81	< 0.0001
Male sex		(All Women)		0.76	0.67 to 0.85	< 0.0001
Age	See Figure 1a		< 0.0001		See Figure 1a	0.0001
Calendar Year	See Figure 1b		< 0.0001		See Figure 1b	< 0.0001
Race/ethnicity (versus Caucasian)						
African American	1.50	1.05 to2.1	0.027	0.86	0.76 to 0.97	0.014
Hispanic	1.64	1.09 to 2.5	0.018	1.44	1.15 to 1.81	0.0018
Other	1.38	0.66 to 2.9	0.40	0.94	0.76 to 1.16	0.57
Area (versus San Francisco)						
Bronx	1.41	0.96 to 2.1	0.080		(All San Francisco area)	
Brooklyn	0.90	0.58 To 1.39	0.63			
Washington DC	1.10	0.70 To 1.73	0.68			
Los Angeles	0.80	0.51 to 1.26	0.33			
Chicago	1.70	1.11 to 2.6	0.015			
HIV infected	1.56	1.16 to 2.1	0.0030	1.27	1.09 to 1.47	0.0017

We examined a number of possible additions or refinements to the models shown. Including blood transfusion history in the WIHS model would result in losing 995 subjects with missing data for this variable, and it did not appear to be an important predictor (OR 1.10, 95% CI 0.74 to 1.65, p = 0.64). Allowing for differing effects of age when reportedly injecting versus not injecting did not produce substantially better fits to the data (WIHS p = 0.38, UHS p = 0.21 by likelihood ratio tests). Other interaction terms examined included calendar year by reported IDU (WIHS p = 0.64, UHS p = 0.39), year by age (WIHS p = 0.37, UHS p = 0.065), race by IDU (WIHS p = 0.13, UHS p = 0.29), and sex by IDU (UHS p = 0.54). We also examined models with IDU effects differing for the 4^th ^to 10^th ^years of reported IDU versus beyond the 10^th ^(WIHS p = 0.33, UHS p = 0.17). We note that the confidence intervals were not narrow enough to rule out potentially important interactions, but in the absence of strong evidence for such interactions we focus on the simpler models without them. One interaction that did reach statistical significance was reported IDU by location in the WIHS study (p = 0.029 overall). This was mainly due to reported IDU being estimated to be less risky in the Bronx (IDU OR's estimated to be smaller by a factor of 0.38, 95% CI 0.16 to 0.91) and Brooklyn (OR's smaller by a factor of 0.42, 95% CI 0.16 to 1.14). The OR's for the main effects of these locations were higher when the interaction was included than those shown in Table 3 (Bronx OR 2.3, Brooklyn OR 1.41). These differences make the Bronx and Brooklyn more similar to the UHS model, with lower OR's for reported IDU and a higher background risk. Nevertheless, the OR for the reported first year of IDU in the Bronx remains nearly 3-fold larger than that in the UHS model. Because we already show details for the UHS model, we do not provide any further details on this WIHS model with interaction terms.

### Reconstruction of yearly past infection probabilities for those infected

**Figure 2:**
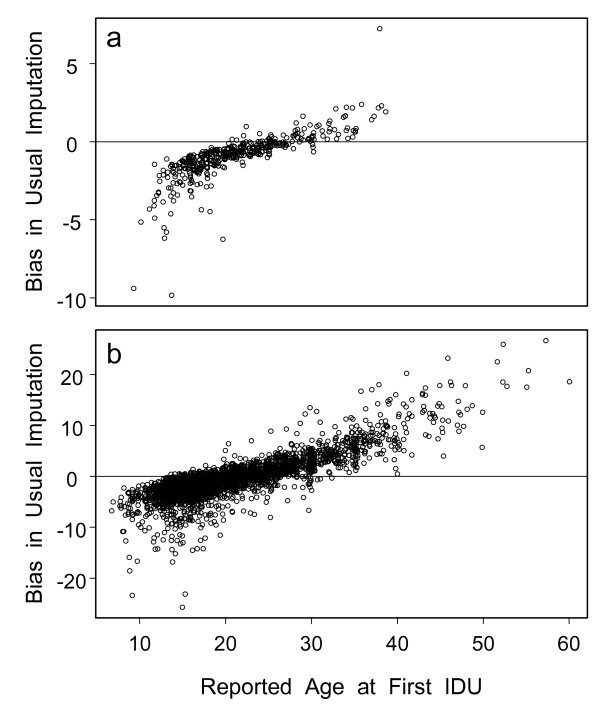
**Estimated biases resulting from imputing age at HCV infection as the age of first IDU**. Bias is defined here as the reported age of first IDU minus the fitted mean from the multivariate models described by Figure 1 and Table 3. Circles below the horizontal line represent subjects who are likely to have been infected after their first IDU, while those above represent subjects likely to have been infected before their first IDU. We have added random numbers ranging from -0.4 to +0.4 to the integer ages in order to increase the visibility of distinct points. Included are HCV seropositive subjects with some history of IDU. **a**) WIHS data, n = 434; **b**) UHS data, n = 4047.

Table 4 summarizes the models' fitted past risks for a number of situations and compares these to the reported age of first IDU, the usual imputed age at HCV infection. Scenarios 1–4 illustrate the impact of different ages at time of HCV antibody test and first IDU, while the remaining scenarios are based on extreme situations observed in the actual data sets. The alternatives at the bottom of the table illustrate the relatively minor impacts of different locations in WIHS and of male sex in UHS. Among all HCV seropositive subjects with IDU history, the median fitted probability that HCV infection occurred the year of first IDU or the next year was 0.77 for WIHS (range 0.23 to 0.93) and 0.56 for UHS (range 0.01 to 0.82). The lower values for UHS reflect its higher estimated background risk when reportedly not injecting and its smaller OR's for the effect of IDU (Table 3). When averaged over all subjects, mean bias was less than 1 year in both studies, because age of reported first IDU was sometimes too early and sometimes too late. There was, however, a strong correlation of bias with reported age of first IDU; the Pearson correlation was 0.78 (95% CI 0.73 to 0.81) in WIHS and 0.83 (95% CI 0.82 to 0.84) in UHS. Figure 2 illustrates this association, showing that those reporting first IDU before age 15 have average predicted ages of infection that are substantially after first IDU, while those reporting first IDU after age 30 have average predicted infection ages that can be many years before first reported IDU.

**Table 4:** Summaries of fits from multivariate models described by Table 3 and Figure 1. Except as noted, area is San Francisco and sex is female.

							WIHS Model			UHS Model		
								
		Age of Infection						Age of Infection			Age of Infection	
								
Scenario	Year of Birth	First IDU	Last IDU	HCV Ab + Test	Daily IDU	Race/Ethnicity	Probability First IDU Accurate*	Fitted Mean	Bias^†^	Probability First IDU Accurate*	Fitted Mean	Bias^†^
1	1960	12	40	40	No	Caucasian	0.38	17.5	-5.5	0.52	15.2	-3.2
2	1940	12	60	60	No	Caucasian	0.07	29.2	-17.2	0.18	22.4	-10.4
3	1960	35	40	40	No	Caucasian	0.66	34.7	0.3	0.39	26.5	8.5
4	1940	35	60	60	No	Caucasian	0.74	35.5	-0.5	0.47	32.3	2.7
5	1943	14	50	52	Yes	Hispanic	0.30	21.9	-7.9	0.58	16.2	-2.2
6	1943	14	50	52	No	Caucasian	0.19	25.4	-11.4	0.33	20.5	-6.5
7	1972	21	22	22	Yes	Hispanic	0.96	21.2	-0.2	0.71	18.5	2.5
8	1977	16	18	18	Yes	Caucasian	0.83	16.7	-0.7	0.81	15.5	0.5
9	1942	56	57	57	Yes	African American	0.09	34.3	21.7	0.07	31.4	24.6
10	1917	15	70	70	Yes	African American	0.01	41.2	-26.2	0.01	39.4	-24.4
2 – LA^‡^	1940	12	60	60	No	Caucasian	0.07	29.6	-17.6			
9 – LA^‡^	1942	56	57	57	Yes	African American	0.09	34.4	21.6			
2 – Ch^§^	1940	12	60	60	No	Caucasian	0.09	28.0	-16.0			
9 – Ch^§^	1942	56	57	57	Yes	African American	0.08	34.0	22.0			
2 – Male	1940	12	60	60	No	Caucasian				0.14	24.1	-12.1
9 – Male	1942	56	57	57	Yes	African American				0.08	32.0	24.0

* Fitted probability that infection occurred in either the year of first IDU or the year after.

^† ^Age at first IDU minus the predicted mean age of infection from the model for that scenario.

^‡ ^Los Angeles area ^§ ^Chicago area

## Discussion

Using modern statistical methods and large data sets, we were able to obtain models for HCV infection risk that can be used to produce year-by-year estimates of past infection risk. Similar to many epidemiological studies, we found high risk in the reported first year of IDU [4,5,7,8,12] and decreasing risk in recent calendar years [22,37-40]. For a patient or research subject newly discovered to be HCV-infected, these models permit calculating the estimated probability that infection occurred at each year in the past based on IDU history and other characteristics. For the subjects studied here, results of such calculations suggest that the common approach of imputing the age of infection as the reported age of first IDU is unreliable.

### Some implications for modeling of fibrosis progression

Although the average difference between reported age of first IDU and the fitted mean infection times from the models was small for both WIHS and UHS, this does not imply that modeling of fibrosis progression will be accurate if it assumes infection at reported age of first IDU. Large errors in one direction cancelled large errors in the other, and there was usually a considerable chance that infection occurred before or after the first reported year of IDU. Such uncertainty necessitates a multiple imputation strategy [41] or other advanced statistical method to avoid both biased estimates and inappropriately narrow confidence intervals. Most importantly, the strong correlation of errors with reported age at first IDU implies that the usual imputation can lead to spurious associations of fibrosis progression with age at first IDU. For example, consider one patient matching scenario 1 in Table 4 who shows Metavir [42] fibrosis stage 4 (cirrhosis) by liver biopsy at age 40, and another patient matching scenario 3 who shows fibrosis stage 2 at age 40. If we calculate progression rate as observed stage divided by duration of infection [14], we get very different comparisons depending on what we assume about age of infection. Using reported age of first IDU, the patient reporting first IDU at age 12 has a rate of 0.143 fibrosis points per year, while the one reporting first IDU at age 35 has a rate of 0.400, nearly three times as fast. But using the fitted means from the UHS model completely eliminates this difference, producing rates of 0.161 and 0.148.

### Additional sources of similar possible bias

The above bias toward making earlier age of HCV infection look spuriously protective against rapid progression is further compounded by other sources of bias. Many clinic-based progression studies have utilized patients whose HCV infection is discovered due to related symptoms. This may exaggerate progression rates compared to the entire population of HCV-infected persons [18], but it can also produce a spurious protective effect of earlier age of infection. Consider 4 persons who all would develop symptoms and have their HCV infection discovered once they reach fibrosis stage 3. Two are fast progressors who reach stage 3 in 15 years, one infected at age 15 and one at age 40. The other two are slow progressors who will reach stage 3 in 45 years, again with one infected at age 15 and one at age 40. Among these 4 persons, progression is not associated with age at infection. Nevertheless, mortality risk unrelated to HCV makes the slow progressor infected at age 40 much less likely than the one infected at age 15 to ever be included in a clinic-based study, while the difference in unrelated mortality risk for the fast progressors is considerably less. This differential selection pressure implies that in clinic-based studies slow progressors will be under-represented among those infected at older ages compared to those infected at younger ages, producing an apparent protective effect of younger age at infection.

An additional difficulty is distinguishing a fixed effect due to age at infection from a time-varying effect that changes as a patient ages. For example, progression may accelerate with increasing age so that the rate of progression is 2-fold faster at age 60 than it would be at age 30, given the same duration of disease and other characteristics. Those infected at younger ages would then have more of their disease course occur at younger ages when progression is slower, causing them to progress more slowly overall even if age at infection itself has no direct effect and even though they experience the same acceleration once they reach older ages. This again could produce a misleading protective effect of younger age at infection. Carefully distinguishing a fixed versus time-varying role for age in fibrosis progression will likely require multi-state modeling algorithms [20] that allow for time-varying covariates, which are currently not available. An analysis of similar issues concerning the role of age in variant Creutzfeldt-Jakob disease [43] required customized analysis to reveal that previous analyses had reached apparently mistaken conclusions. Similar efforts may be worthwhile for HCV.

### Limitations

The current status data available in this cross-sectional study are less informative than knowing exact times of HCV infection from a longitudinal study, but the large sample sizes allowed our methods to produce models with many plausible features, reasonably narrow confidence intervals, and many small p-values. Importantly, this cross-sectional situation matches that of many progression studies where subjects are found to be chronically HCV infected without any direct information on when they became infected [14-21]. Although potentially counterintuitive, this type of data does permit estimation of effects of calendar time before the year of the first HCV antibody test and of the background risk without IDU despite the lack of anyone with no IDU in the UHS study. This is because the current status data provide information about cumulative risk over all years before the measurement of the outcome, and because we had considerable variation in the ages, calendar years, and reported durations of IDU at the time of HCV antibody testing. For example, information on background risk when reportedly not injecting can be obtained by comparing the HCV prevalence of persons with 1 year of IDU tested at age 20 versus persons with 1 year of IDU tested at age 40, because they have different amounts of non-IDU time. This comparison also provides information on the risk of the first year of IDU, because this is the risk the two groups have in common after the difference in background risk is accounted for. Our analyses synthesize many such comparisons to produce the estimates.

The self-reported risk factor histories we have used may be inaccurate, due to inaccurate recall or social desirability bias. Indeed, some (or most) of the apparent background risk of infection in the absence of IDU could be due to inaccurate recall or report of ages of IDU. Our results therefore do not necessarily imply substantial risk from sources other than IDU and therefore should not be interpreted as contradicting the established belief that IDU is the overwhelmingly predominant source of chronic HCV infections. Some of the 8.6% prevalence of HCV seropositivity among WIHS subjects reporting no IDU could reflect infections caused by forgotten or unreported IDU. More importantly, the estimated risk in the absence of IDU is very high in UHS, much higher than in WIHS, reaching incidence of 3.4% per year for the riskiest calendar year and riskiest age. This is reflected in Figure 2b, where 31% of the predicted mean infection ages are before the reported age of first IDU (points above the horizontal line). Utilizing the entire fitted distributions instead of just the predicted means, we obtained an estimate of 17% of UHS subjects being infected before their reported age of first IDU. Although there is likely to be some risk in the absence of IDU, particularly in this high-risk population, and one study found a similarly high prevalence of HCV infection among users of non-injection drugs [44], we believe that much of the risk predicted by the model may reflect inaccurate reporting of ages of first IDU. Thus, our models' fitted background risks may reflect risk in the absence of *reported *IDU, but they may be inaccurate estimates of risk when there really is no IDU. Fortunately, this fits with our primary purpose of studying imputation of age of infection based on *reported *risk factor history, which is what must often be done in studies of fibrosis progression. For this purpose, it is irrelevant whether risk of infection before reported age of first IDU results from non-IDU sources of risk or from unreported IDU. In either case, imputation may be inaccurate.

One aspect that does not match the situation in progression studies is the use of HCV serology as the outcome. The reconstructed yearly risks summarized in Table 4 and Figure 2 are conditional on HCV seropositivity instead of being conditional on confirmed chronic HCV infection (by HCV RNA), which will be the case in progression studies. The proportion of HCV seropositive persons who clear the virus and become HCV RNA- is thought to be about 20% overall [45] and 15% among HIV-infected persons [46]. A model of clearance probabilities utilizing age and other characteristics could in principle be synthesized with the models presented here to produce more relevant reconstructed past infection probabilities that are conditional on chronic HCV infection. In addition, some chronically HCV infected persons may test HCV seronegative, particularly by the second generation assay mainly used here. In one study of HIV-infected persons, 37 of 617 (6%) with HCV infection were observed to be HCV negative by second generation antibody assay [46]. We note that some of these 37 cases may have been recently infected and not yet produced antibodies, which would not be typical of subjects in HCV progression studies. Exposure to HCV that results in neither chronic infection nor HCV seropositivity [47] does not affect the validity of our results, because such events do not initiate progression of HCV-related liver damage and are correctly treated as such in our analyses.

The necessary exclusion of persons who died before the conduct of the study could result in biased estimates of risk factors, and any such "survivor bias" may be worse in the WIHS, where participants also had to survive to the visit where IDU history was assessed. For example, if those who die of overdose in the first year of IDU are much more likely to have been HCV infected, then their exclusion will result in an underestimate of the HCV risk during the first year of IDU. We note, however, that cross-sectional progression studies will also necessarily have excluded persons who die before being tested for HCV. Thus, our estimates remain relevant for our primary purpose of assessing estimation of past infection times in such studies.

Being HIV positive was included as a fixed risk factor in multivariate models, because the timing of HIV infection was not known for most HIV-infected subjects. This assumption could make sense if HCV risk associated with being HIV infected was mainly due to other risk factors (such as membership in a risky social network) that are not directly available for modeling but are associated with HIV infection and were present even before HIV infection. If the mechanism of HIV-associated risk is directly causal, such as greater susceptibility to HCV when HIV infection is already present, then this effect would not be present before HIV infection occurred. In this case, our estimates based on HIV as a fixed risk factor would be attenuated compared to what would be estimated if the timing of past HIV infection were known. Although IDU might be expected to usually result in HCV infection before it causes HIV infection, sexual HIV risk is important among drug users [26], so HIV could precede HCV infection often enough that the possibility of attenuation cannot be ruled out.

Despite many qualitative similarities, the differences between the WIHS and UHS models leave considerable uncertainty about how to impute ages of infection for a progression study. Because inclusion of WIHS participants required retention in the study until IDU history was assessed, these may be less similar than UHS participants to cross-sectional progression study participants, while perhaps being more similar to subjects who are retained in longitudinal follow-up. A desirable design would be a large cross-sectional study ascertaining chronic HCV infection and risk factor histories, followed by fibrosis progression measurement in those found to be HCV-infected. Infection models fit to the cross-sectional data would then be directly applicable to the progression modeling. (The *most *desirable design, prospectively ascertaining incident infections and subsequently monitoring progression, would likely be prohibitively difficult.) In the absence of such directly relevant models of past risk, a sensible strategy may be to perform sensitivity analyses using both models presented here, along with the usual imputation based on first year of IDU. In addition, within each model different specific choices about whether to include and how to model each predictor, as well as whether to include some interaction terms, could in some cases be reasonable. This adds additional uncertainty about specific imputations, but additional uncertainty only adds further support for our conclusion that the common single-imputation strategy is dangerous.

## Conclusion

We have shown that, in cross-sectional HCV progression studies that rely on risk factor histories to impute time of HCV infection, there is usually considerable uncertainty about when HCV infection may have occurred, even for patients or research subjects reporting a history of IDU. This should be accounted for in such progression studies, preferably using modern methods such as multiple imputation [41]. To facilitate use of such methods, we provide, in Additional File 2, code that produces individuals' past infection probabilities year by year. Use of single imputation can not only produce confidence intervals that are too narrow and p-values that are too small, but also severely biased estimates of covariate effects. In particular, the usual strategy of imputing age of HCV infection as the age of first reported IDU is likely to produce bias toward finding slower progression associated with younger age of infection. This bias could be further exacerbated by differential selection effects according to age of infection, and by time-varying effects of current age being mis-modeled as fixed effects of age at infection. Some or all of the protective effect of younger age at infection [14,19] found in cross-sectional studies of fibrosis progression could therefore be spurious.

## Abbreviations used

CI – confidence interval

HCV – hepatitis C virus

HIV – human immunodeficiency virus

IDU – injection drug use

OR – odds ratio

RNA – ribonucleic acid

UHS – San Francisco Urban Health Study

WIHS – Women's Interagency HIV Study

## Competing interests

The author(s) declare that they have no competing interests.

## Authors' contributions

PB conceived of the study, performed all analyses, and drafted and revised the manuscript. PCT, ECS, and MHA participated in the performance of the WIHS study and revised the manuscript for critical scientific content. TRO and MPB promoted and facilitated the performance of HCV testing in the UHS and revised the manuscript for critical scientific content. AHK and BRE led the performance of the UHS study, and revised the manuscript for critical scientific content. In addition, BRE raised issues leading to the conception of this study. All authors read and approved the final manuscript.

## Pre-publication history

The pre-publication history for this paper can be accessed here:



## Supplementary Material

Additional file 1 **SAS code for fitting a model**. This gives the NLMIXED command used to fit the WIHS model described by Table 3 and Figure 1. Click here for file

Additional file 2 **R/S-plus code for obtaining conditional densities**. This gives code that will produce fitted probabilities for HCV infection having occurred at each year of age up to the age of a positive HCV antibody test, based on subject characteristics and IDU history. Click here for file
