# Supplementary material for: Estimating past hepatitis C infection risk from reported risk factor histories: implications for imputing age of infection and modeling fibrosis progression
Source: BMC Infect Dis. 2007 Dec 10;7:145. doi: 10.1186/1471-2334-7-145 (PMC2238758; doi:10.1186/1471-2334-7-145)
Supplement: Additional file 1 — SAS code for fitting a model. This gives the NLMIXED command used to fit the WIHS model described by Table 3 and Figure 1. [file 1471-2334-7-145-S1.pdf]

```
* Data set contains variables:
* hcv       1 if anti-hcv positive, 0 if negative
* calcbyr   year of birth
* first     age first at risk of HCV; 1 in all cases
* last      age of anti-hcv antibody test
* agefinj   reported age of first injection drug use, last+1 if none
* agelinj   reported age of last injection drug use, last if ongoing
* daily     1 if typical frequency of injection was at least daily, 0 if less
* black     1 if African American, 0 otherwise
* latino    1 if Hispanic, 0 otherwise
* othrace   1 if not Caucasian, AA, or Hispanic, 0 otherwise
* Bronx     1 if WIHS site was the Bronx, 0 otherwise
* Brooklyn  1 if WIHS site was Brooklyn, 0  otherwise
* dc        1 if WIHS site was Washington, DC, 0  otherwise
* la        1 if WIHS site was Los Angeles, 0  otherwise
* Chicago   1 if WIHS site was Chicago, 0  otherwise
* hiv       1 if HIV positive, 0 if negative ;

proc nlmixed cov tech=nrridg absgconv=1e-9[5] maxiter=100 maxfunc=10000;
* starting values for parameters ;
 parms intercept=-5 inj1=0 inj23=0 inj4up=0
   age1=0 age2=0 dailyuse=0 year=0 year2=0
   blackk=0 latin=0 othrac=0 brnx=0 brook=0 atdc=0 atla=0 chicag=0
   hivpos=0;
 surv=1;  * surv is the probability of remaining anti-HCV negative ;
* The following contribution to risk applies to every age ;
 baselogit=intercept+blackk*black+latin*latino+othrac*othrace +hiv*hivpos
      + bronx*brnx + brooklyn*brook + dc*atdc + la*atla + chicago*chicag;
 do age=first to last; * accumulate risk over ages up to anti-HCV test ;
   yr=calcbyr+age-2000;  * calendar year, relative to 2000 ;
   * add risk contributions due to age and calendar year ;
   logithaz=baselogit+ age1*age+age2*age*age + year*yr+year2*yr*yr;
   if age<agefinj | age>agelinj then chaz=1/(1+exp(logithaz));
   * chaz is 1 – hazard ;
   injterm=dailyuse*daily;
   * Assume IDU started halfway through agefinj ;
   if age=agefinj then do;
     chaz1=sqrt(1/(1+exp(logithaz))); * first half of age, no IDU ;
       chaz2=sqrt(1/(1+exp(logithaz+inj1+injterm))); * IDU in 2nd half ;
       chaz=chaz1*chaz2;
   end;
   if age=agefinj+1 & age<=agelinj then do;  * Similar calculations ;
       chaz1=sqrt(1/(1+exp(logithaz+inj1+injterm)));
       if age<agelinj then chaz2=sqrt(1/(1+exp(logithaz+inj23+injterm)));
       if age=agelinj then chaz2=sqrt(1/(1+exp(logithaz)));
       chaz=chaz1*chaz2;
   end;
   if age=agefinj+2 & age<=agelinj then do;  * Similar calculations ;
       chaz1=sqrt(1/(1+exp(logithaz+inj23+injterm)));
       if age<agelinj then chaz2=sqrt(1/(1+exp(logithaz+inj23+injterm)));
       if age=agelinj then chaz2=sqrt(1/(1+exp(logithaz)));
       chaz=chaz1*chaz2;
   end;
   if age=agefinj+3 & age<=agelinj then do;  * Similar calculations ;
       chaz1=sqrt(1/(1+exp(logithaz+inj23+injterm)));
       if age<agelinj then chaz2=sqrt(1/(1+exp(logithaz+inj4up+injterm)));
       if age=agelinj then chaz2=sqrt(1/(1+exp(logithaz)));
```

```
        chaz=chaz1*chaz2;
    end;
    if agefinj+3<age & age<=agelinj then do;  * Similar calculations ;
        chaz1=sqrt(1/(1+exp(logithaz+inj4up+injterm)));
        if age<agelinj then chaz2=sqrt(1/(1+exp(logithaz+inj4up+injterm)));
        if age=agelinj then chaz2=sqrt(1/(1+exp(logithaz)));
        chaz=chaz1*chaz2;
    end;
    * adjust risk for the age when the test occurred ;
    if age=last then do; if agefinj<age<=agelinj then chaz=chaz1;
    else chaz=sqrt(chaz); end;
    surv=surv*chaz; * track the accumulating risk ;
end;  * end of loop through all years at risk ;
 p=1-surv; * probability of anti-HCV+ ;
 model hcv ~ binary(p);
 title 'WIHS, quadratic calendar year and age';
 run;
```
