# Supplementary material for: Estimating past hepatitis C infection risk from reported risk factor histories: implications for imputing age of infection and modeling fibrosis progression
Source: BMC Infect Dis. 2007 Dec 10;7:145. doi: 10.1186/1471-2334-7-145 (PMC2238758; doi:10.1186/1471-2334-7-145)
Supplement: Additional file 2 — R/S-plus code for obtaining conditional densities. This gives code that will produce fitted probabilities for HCV infection having occurred at each year of age up to the age of a positive HCV antibody test, based on subject characteristics and IDU history. [file 1471-2334-7-145-S2.pdf]

Below is code that runs in either R (http://CRAN.R-project.org/) or S-plus
(http://www.insightful.com/products/splus/) statistical packages. Use the Select Tool in Adobe
Reader to copy this and paste it into either S-plus or R. For someone with a positive test for HCV infection,
this returns fitted probabilities for when he or she was infected, based on information supplied in the vector
"preds".

```
library("splines")  # Include this if running in R
wihsParams <- c(
-12.592143883, #  1  intercept
  5.736048710, #  2      inj1
  5.091142888, #  3     inj23
  1.972396705, #  4    inj4up
  0.127726176, #  5  dailyuse
  0.348384142, #  6      ageLinear
 -0.005591044, #  7      ageQuadratic
 -0.163715768, #  8      yearLinear
 -0.003514481, #  9     yearQuadratic
  0          , # 10      yearSpline1
  0          , # 11      yearSpline2
  0          , # 12      yearSpline3
  0          , # 13      yearSpline4
  0          , # 14      man
  0.403782049, # 15    blackk
  0.492086770, # 16     latin
  0.323003435, # 17    othrac
  0.345907244, # 18      brnx
 -0.107515819, # 19     brook
  0.095883287, # 20      atdc
 -0.222709684, # 21      atla
  0.529358756, # 22    chicag
  0.443385834) # 23    hivpos


uhsParams <- c(
-14.925894242, #  1 intercept
  4.112592320, #  2    inj1
  2.284821603, #  3   inj23
  1.648190843, #  4  inj4up
  0.463629622, #  5 dailyuse
  0.152336572, #  6    ageLinear
 -0.002491117, #  7    ageQuadratic
  0          , #  8   yearLinear
  0          , #  9   yearQuadratic
  8.224213751, # 10    yearSpline1
  4.848163946, # 11    yearSpline2
 16.663527041, # 12    yearSpline3
 -0.866023880, # 13    yearSpline4
 -0.275295695, # 14      man
 -0.150010414, # 15   blackk
  0.364329332, # 16    latin
 -0.061395638, # 17   othrac
  0          , # 18      brnx
  0          , # 19     brook
  0          , # 20      atdc
```

```
  0          , # 21      atla
  0          , # 22    chicag
  0.236203407) # 23   hivpos

yr4 <- ns(1917:2003,knots=c(1980,1990,1995))

conddens <- function(preds, params) {

  intercept <- params[1]
  inj1 <- params[2]
  inj23 <- params[3]
  inj4up <- params[4]
  dailyuse <- params[5]
  ageLinear <- params[6]
  ageQuadratic <- params[7]
  yearLinear <- params[8]
  yearQuadratic <- params[9]
  yearSpline1 <- params[10]
  yearSpline2 <- params[11]
  yearSpline3 <- params[12]
  yearSpline4 <- params[13]
  man <- params[14]
  blackk <- params[15]
  latin <- params[16]
  othrac <- params[17]
  brnx <- params[18]
  brook <- params[19]
  atdc <- params[20]
  atla <- params[21]
  chicag <- params[22]
  hivpos <- params[23]

# The block below specifies the covariate values that must be specified in order to
# estimate a person’s past year-by-year risk of HCV infection from this model

  first <- preds[1]      # Age first at risk.  This will be 1 for current status data.
  last <- preds[2]       # Age of HCV test result
  calcbyr <- preds[3]    # Calendar year of birth
  agefinj <- preds[4]    # Age of first IDU, set to last+1 if none
  agelinj <- preds[5]    # Age of last IDU (can be missing if agefinj>last)
  daily  <- preds[6]     # 1 if daily IDU when injected, 0 otherwise
  male <- preds[7]       # 1 if male, 0 if female
  black <- preds[8]      # 1 if black race, 0 otherwise
  latino <- preds[9]     # 1 if Hispanic, 0 otherwise
  othrace <- preds[10]   # 1 if race/ethnicity other than Caucasian, black, Hispanic
  bronx <- preds[11]     # 1 if WIHS Bronx site, 0 otherwise
  brooklyn <- preds[12]  # 1 if WIHS Brooklyn site, 0 otherwise
  dc <- preds[13]        # 1 if WIHS Washington, DC site, 0 otherwise
  la <- preds[14]        # 1 if WIHS Los Angeles site, 0 otherwise
  chicago <- preds[15]   # 1 if WIHS Chicago site, 0 otherwise
  hiv <- preds[16]       # 1 if HIV infected, 0 if not

       baselogit <- intercept+blackk*black+latin*latino+othrac*othrace+male*man+
          bronx*brnx + brooklyn*brook + dc*atdc + la*atla + chicago*chicag+hivpos*hiv
      chaz <- numeric()  # chaz is (1 - hazard)

 for (age in (first:last)) {
```

```
    yr <- calcbyr+age-2000
    logithaz <- baselogit + ageLinear*age+ageQuadratic*age*age + yearLinear*yr +
             yearQuadratic*yr*yr +yearSpline1*yr4[yr+84,1]+yearSpline2*yr4[yr+84,2]+
             yearSpline3*yr4[yr+84,3]+yearSpline4*yr4[yr+84,4]
   if ((age<agefinj) | (age>agelinj)) chazup <- 1/(1+exp(logithaz))
    injterm <- dailyuse*daily
    if (age==agefinj) {
      chaz1 <- sqrt(1/(1+exp(logithaz)))
        chaz2 <- sqrt(1/(1+exp(logithaz+inj1+injterm)))
        chazup <- chaz1*chaz2
    }
    if ((age==(agefinj+1)) & (age<=agelinj)) {
        chaz1 <- sqrt(1/(1+exp(logithaz+inj1+injterm)))
        if (age<agelinj) chaz2 <- sqrt(1/(1+exp(logithaz+inj23+injterm)))
        if (age==agelinj) chaz2 <- sqrt(1/(1+exp(logithaz)))
        chazup <- chaz1*chaz2
    }
    if ((age==(agefinj+2)) & (age<=agelinj)) {
        chaz1 <- sqrt(1/(1+exp(logithaz+inj23+injterm)))
        if (age<agelinj)  chaz2 <- sqrt(1/(1+exp(logithaz+inj23+injterm)))
        if (age==agelinj) chaz2 <- sqrt(1/(1+exp(logithaz)))
        chazup <- chaz1*chaz2
    }
    if ((age==(agefinj+3)) & (age<=agelinj)) {
        chaz1 <- sqrt(1/(1+exp(logithaz+inj23+injterm)))
        if (age<agelinj) chaz2 <- sqrt(1/(1+exp(logithaz+inj4up+injterm)))
        if (age==agelinj) chaz2 <- sqrt(1/(1+exp(logithaz)))
        chazup <- chaz1*chaz2
    }
    if (((agefinj+3)<age) & (age<=agelinj)) {
        chaz1 <- sqrt(1/(1+exp(logithaz+inj4up+injterm)))
        if (age<agelinj) chaz2 <- sqrt(1/(1+exp(logithaz+inj4up+injterm)))
        if (age==agelinj) chaz2 <- sqrt(1/(1+exp(logithaz)))
        chazup <- chaz1*chaz2
    }
    if (age==last) {
             if ((agefinj<age) & (age<=agelinj)) chazup <- chaz1
             if ((agefinj>=age) | (age>agelinj)) chazup <- sqrt(chazup)
       }
       chaz  <-  c(chaz,chazup)
} # end of age loop
surv  <-  cumprod(chaz)
surv  <-  c(1,surv[1:(length(surv)-1)])
dens  <-  (1-chaz)*surv
cbind(age=(first:last),cdens=(dens/sum(dens)))
}

#  BELOW IS AN EXAMPLE, CORRESPONDING TO THE FIRST 4 LINES OF TABLE 4

tab4data <- rbind(
c(1,40,1960,12,40,rep(0,11)),
c(1,60,1940,12,60,rep(0,11)),
c(1,40,1960,35,40,rep(0,11)),
c(1,60,1940,35,60,rep(0,11)))

wihsdens <- apply(tab4data,1,conddens,params=wihsParams)
wihsdens <- as.array(wihsdens)
```

```
uhsdens <- apply(tab4data,1,conddens,params=uhsParams)
uhsdens <- as.array(uhsdens)

wihsCmean <- numeric()
wihsprob2 <- numeric()
uhsCmean <- numeric()
uhsprob2 <- numeric()
for (i in 1:4) {
      finj <- tab4data[i,4]
      densup <- wihsdens[[i]]
      wihsCmean <- c(wihsCmean,sum(densup[,1]*densup[,2]))
      wihsprob2 <- c(wihsprob2,sum(densup[c(finj,finj+1),2]))
      densup <- uhsdens[[i]]
      uhsCmean <- c(uhsCmean,sum(densup[,1]*densup[,2]))
      uhsprob2 <- c(uhsprob2,sum(densup[c(finj,finj+1),2]))
}
round(cbind(wProb2=wihsprob2,wMean=wihsCmean,wBias=tab4data[,4]-wihsCmean,
      uProb2=uhsprob2,uMean=uhsCmean,uBias=tab4data[,4]-uhsCmean),2)

# OUTPUT SHOULD BE AS BELOW:
#
#       wProb2 wMean  wBias uProb2 uMean  uBias
# [1,]   0.38 17.47  -5.47   0.52 15.17  -3.17
# [2,]   0.07 29.25 -17.25   0.18 22.44 -10.44
# [3,]   0.66 34.75   0.25   0.39 26.55   8.45
# [4,]   0.74 35.54  -0.54   0.47 32.32   2.68
```
